# Effect of post‐harvest ultrasound treatment on phytochemical enhancement in *Gynura procumbens* leaves and their protection against oxidative stress‐induced muscle atrophy

**DOI:** 10.1002/jsfa.70335

**Published:** 2025-11-20

**Authors:** Taemin Jeong, Seonghwa Hong, Jeehye Sung, Heon‐Sang Jeong, Hana Lee, Junsoo Lee

**Affiliations:** ^1^ Department of Food Science and Biotechnology Chungbuk National University Cheongju Korea; ^2^ Department of Food Science and Biotechnology Gyeongkuk National University Andong Korea

**Keywords:** sarcopenia, muscle atrophy, *Gynura procumbens*, ultrasound, metabolomics

## Abstract

**BACKGROUND:**

*Gynura procumbens* is a medicinal plant widely recognized for its antioxidant and anti‐inflammatory properties. Despite its traditional use in Southeast Asia, its potential role in promoting muscle health has not been thoroughly investigated. Sarcopenia, characterized by the progressive loss of muscle mass and strength during aging, remains a significant public health concern with limited therapeutic options. This study aimed to enhance the phytochemical content of *G. procumbens* leaves through ultrasound treatment and to evaluate the biological effects of treated extracts on muscle atrophy in a C2C12 myoblast and myotube model.

**RESULTS:**

Ultrasound treatment for 10 min significantly increased the activities of catalase, peroxidase, phenylalanine ammonia‐lyase, and tyrosine ammonia‐lyase. These enzymatic changes were associated with elevated levels of phenolic acids and flavonoids, including quercetin, rutin, and kaempferol. The 10 min ultrasound‐treated *G. procumbens* (UTGP) extract enhanced cell viability under oxidative stress conditions and promoted the expression of myogenic differentiation markers in C2C12 cells. Targeted and untargeted metabolomics analyses confirmed the accumulation of key bioactive compounds and their strong correlation with muscle‐protective activities.

**CONCLUSIONS:**

Ultrasound treatment is an effective postharvest strategy to enhance the phytochemical profile and functional properties of *G. procumbens*. The 10 min UTGP extract demonstrated significant potential for preventing muscle atrophy by improving cellular antioxidant capacity and promoting muscle differentiation. These findings provide a scientific basis for the development of *G. procumbens*‐based functional foods or nutraceuticals targeting age‐related muscle loss. © 2025 The Author(s). *Journal of the Science of Food and Agriculture* published by John Wiley & Sons Ltd on behalf of Society of Chemical Industry.

## INTRODUCTION

Ultrasound is a sound wave with frequencies greater than 20 kHz which is above the upper limit of human hearing. Ultrasound is widely used in many different fields including the food industry and is considered safe, environmentally friendly, and non‐toxic.^1^ A recent study has shown that ultrasound induces water molecules to split and generate highly active free radicals, thus producing and increasing reactive oxygen species (ROS) and causing oxidative damage in plant cells.^2^ Ultrasound reportedly acts as an abiotic elicitor, inducing the plant defense mechanism and resulting in altered enzyme activities associated with phytochemical biosynthesis pathways.^3^, ^4^ While ultraviolet (UV) or light‐emitting diode (LED) light primarily interacts with photoreceptors located on the plant surface, ultrasound can penetrate deeper into plant tissues and often requires shorter exposure times. This enables ultrasound to induce more extensive defense responses, resulting in increased production and accumulation of secondary metabolites that provide various health benefits.^5^ It has been demonstrated that ultrasound treatment increases the levels of flavonoids, including daidzein and genistein in soybean sprouts.^6^ Moreover, phytochemicals, including triterpenes, in *Centella asiatica* leaves are enhanced by ultrasound treatment.^3^ Therefore, as a postharvest treatment for plants, ultrasound can enhance the accumulation of functional compounds and ultimately increase various biological activities.

With rising life expectancy, population aging has become the most pervasive and dominant global demographic trend, with the progression of large cohorts into older ages of 65 years and above.^7^ The rise in life expectancy is inversely correlated with an increase in the incidence of age‐related diseases, including cardiovascular disorders, neurodegenerative conditions, type 2 diabetes, osteoporosis, and sarcopenia.^8^ Especially, with advancing age, a sequential decline in skeletal muscle mass, function, and regenerative capacity has been noted.^9^ This decline is linked to elevated levels of ROS *in vivo*, which exacerbate apoptosis and cellular mortality and hinder myoblast differentiation, thereby impairing muscle repair processes. The development of sarcopenia involves several key parameters, including the disruption of muscle cell regeneration, elevated cell death, mitochondrial dysfunction, and disturbances in protein metabolism.^10^ Moreover, disruption of the redox balance leads to increased oxidative stress, which results in lipid peroxidation of skeletal muscle.^11^ Therefore, the development of effective therapeutic materials that are specifically designed to prevent and treat these conditions is crucial to reduce their impact on the aging population and improve the overall quality of life.

*Gynura procumbens* belongs to the Asteraceae family and has significant medicinal value in Southeast Asia, particularly in Thailand, Indonesia, and Malaysia.^12^ The leaves of *G. procumbens* are ethnopharmacologically used to regulate blood glucose levels and treat inflammation, viral ailments, rheumatic fever, and kidney discomfort.^13^ Hassan *et al*.^14^ showed that the glucose uptake and utilization increased when *G. procumbens* extracts were administered. Poh *et al*.^15^ reported that *G. procumbens* may counteract the vasoconstrictive effects of angiotensin II via endothelium‐dependent mechanisms. In addition, Nisa *et al*.^16^ demonstrated that *G. procumbens* has potential for cancer prevention. These actions have been attributed to the presence of various chemical constituents, such as phenolic acids, flavonoids, saponins, tannins, terpenoids, and alkaloids.^17^ Notably, the major flavonoids in *G. procumbens* leaves are quercetin and kaempferol.^12^, ^18^ Despite the presence of various functional compounds, the effects of *G. procumbens* leaves on the improvement of muscle atrophy have not yet been fully elucidated.

Therefore, this study aimed to comprehensively evaluate the effects of ultrasound treatment on the biological activities of *G. procumbens* leaves. Specifically, we investigated whether ultrasound treatment enhances the accumulation of secondary metabolites in *G. procumbens* leaves and whether ultrasound‐treated *G. procumbens* (UTGP) leaves exhibits muscle‐protective effects in C2C12 myoblasts and myotubes. Furthermore, untargeted metabolomic profiling was conducted to identify metabolite alterations induced by ultrasound treatment, followed by targeted metabolomic analysis to validate key metabolites. Finally, correlation analysis was performed to predict potential bioactive compounds associated with the observed muscle‐protective effects.

## MATERIALS AND METHODS

### Sample preparation

The leaves of *G. procumbens* were obtained from a local market in Korea, and samples were stored at 4 °C. Subsequently, a sample (100 g) was transferred into an ultrasonic chamber (40 kHz) and treated with ultrasound at 300 W and for 5, 10, and 20 min at 25 °C. UTGP leaves were then lyophilized and stored in a deep freezer until further analysis. For analysis, 5 g of non‐treated and UTGP leaves were extracted using methanol. The extracts were filtered and evaporated, and the dried residues were dissolved in dimethyl sulfoxide.

### Measurement of total phenolic and total flavonoid contents and antioxidant activities

The total phenolic content (TPC), total flavonoid content (TFC), and antioxidant activities based on 2,2‐diphenyl‐1‐picrylhydrazyl (DPPH) and 2,2′‐azino‐bis(3‐ethylbenzothiazoline‐6‐sulfonic acid (ABTS) radical scavenging activities and reducing power of UTGP leaves were determined using colorimetric methods as previously described.^19^

### Activity assays of catalase, peroxidase, phenylalanine ammonia‐lyase, and tyrosine ammonia‐lyase

UTGP leaves were homogenized in 5 mL of phosphate buffer (10 mmol/L, pH 7.4). The homogenate was centrifuged at 14 000 × g for 15 min, and the supernatant was collected for further analysis.^3^ To determine the catalase (CAT) activity assay, 250 μL of the extract was mixed with 2.5 mL of phosphate buffer (10 mmol/L, pH 7.4) and 200 μL of hydrogen peroxide (H_2_O_2_). To examine the peroxidase (POD) activity 100 μL of the extract was combined with 3 mL of phosphate buffer (0.05 mol/L, pH 6.0), 150 mmol/L guaiacol, and 200 μL of H_2_O_2_. The enzymatic activity was measured using a spectrophotometer at 240 and 450 nm for CAT and POD, respectively, over a duration of 30 min. The results are expressed in units per gram of dry weight (U/g DW). The activities of phenylalanine ammonia‐lyase (PAL) and tyrosine ammonia‐lyase (TAL) in the UTGP leaves were determined using previously established protocols.^20^

### Protective effect in C2C12 myoblasts and myotubes

The C2C12 cells were obtained from ATCC and seeded in 96‐well plates at a density of 5.0 × 10^4^ cells/mL. After 24 h, the cells were treated with samples at various concentrations (25, 50, and 100 μg/mL) to evaluate cytotoxicity. To induce oxidative stress, H_2_O_2_ was applied at concentrations ranging from 400 to 700 μmol/L. Based on the preliminary experiments, 50 μg/mL UTGP and 500 μmol/L H_2_O_2_ were selected. After 2 h of UTGP leaves (50 μg/mL) treatment, the culture medium was replaced with H_2_O_2_‐containing medium, and cells were incubated for 24 h. After incubation, 20 μL of 3‐(4,5‐dimethylthiazol‐2‐yl)‐2,5‐diphenyltetrazolium bromide (5 mg/mL) was added to each well and incubated for 2 h. The supernatant was removed, and the blue formazan crystals produced in the viable cells were dissolved in 200 μL of dimethyl sulfoxide. Myoblasts were differentiated into myotubes by culturing in Dulbecco's modified Eagle medium (DMEM) supplemented with 2% horse serum for 6 days. After differentiation, the protective effects were assessed using the same experimental procedures as those employed for the myoblast experiments.

### Measurement of glutathione, malondialdehyde, superoxide dismutase activity, catalase activity, and ROS production in myoblasts

Myoblasts were seeded in six‐well plates at a density of 1.5 × 10^5^ cells/mL. After 24 h, the medium was replaced with a serum‐free medium containing UTGP leaf extracts (50 μg/mL). After 4 h, the cells were treated with 500 μmol/L of H_2_O_2_ for 24 h to induce oxidative damage. To measure the glutathione (GSH) levels, 20 μL of the supernatant was added to 180 μL of a mixture containing GSH reductase, nicotinamide adenine dinucleotide phosphate, and 5,5′‐dithiobis(2‐nitrobenzoic acid). The malondialdehyde (MDA) levels were measured using a thiobarbituric acid reactive substance assay. The superoxide dismutase (SOD) activity was evaluated by measuring the reduction of 2,3‐bis‐(2‐methoxy‐4‐nitro‐5‐sulfophenyl)‐2H‐tetrazolium‐5‐carboxanilide mediated by xanthine/xanthine oxidase. To determine the CAT activity, the amount of H_2_O_2_ remaining in the sample after CAT action was measured. For ROS measurements, the fluorescence intensity of dichlorofluorescein was assessed using flow cytometry and confocal microscopy.

### Measurement of diameter, myosin heavy chain area, and fusion index in myotubes

The C2C12 cells were seeded on a 24‐well plate with coverslips at a density of 1 × 10^5^ cells/mL and differentiated using 2% horse serum for 6 days. After differentiation, the medium was replaced with a serum‐free medium containing 50 μg/mL of the UTGP extract. After 2 h, the cells were co‐treated with samples and 500 μmol/L of H_2_O_2_ for 24 h to induce muscle injury. Myotubes were washed with phosphate‐buffered saline (PBS) and fixed in 4% formaldehyde for 20 min at room temperature. The fixed myotubes were permeabilized in 0.3% Tween 20 for 15 min and blocked with 2% bovine serum albumin for 1 h. The myotubes were then incubated with myosin heavy chain (MHC) antibody (1:100) overnight at 4 °C. The following day, the myotubes were incubated for 1 h with a secondary antibody and then incubated with 4′,6‐diamidino‐2‐phenylindole (DAPI, 0.1 μg/mL) for nuclear staining. The myotubes on the coverslips were observed and photographed (five random areas) using a confocal laser scanning microscope with a 10× lens (LSM‐880 with Airyscan, ZEISS, Oberkochen, Germany). MHC‐positive area (%) was determined as the area of MHC expression (green). The fusion index was calculated as the percentage of nuclei within the MHC‐positive myotubes with at least two nuclei relative to the total number of nuclei. The diameter was determined using ImageJ software.

### Untargeted analysis using liquid chromatography–tandem mass spectrometry

For untargeted metabolomic analysis, 50 mg of lyophilized *G. procumbens* leaves were placed in a centrifuge tube, and 500 μL of precooled methanol was added as the extraction solvent. The mixture was vortexed vigorously for 10 min, followed by sonication in an ice‐cooled ultrasonic bath for 60 min. After centrifugation at 12 000 × *g* for 10 min at 4 °C, the supernatant was collected and filtered through a 0.22 μm nylon membrane. Chromatographic separation was performed using a Thermo Vanquish (Thermo Fisher Scientific, Waltham, MA, USA). A Waters Cortecs T3 column (150 mm × 2.1 mm, 1.6 μm) was used for separation; the column temperature was maintained at 45 °C. The mobile phase was composed of water with 0.1% formic acid (phase A) and acetonitrile with 0.1% formic acid (phase B). The gradient elution program was as follows: 0–15 min, 3–15% B; 15–50 min, 15–100% B; 50–55 min, 100% B; 55–60 min, 100–3% B. The flow rate was set to 0.25 mL/min. A Thermo Q‐Exactive mass spectrometer (Thermo Fisher Scientific) was used for detection. The parameters for the heated‐electrospray ionization source were set based on experience, as follows: ion transfer tube temperature, 320 °C; sheath gas flow, 50 arbitrary units (arb); auxiliary gas flow, 10 arb; and sweep gas flow, 1 arb. The mass spectra were obtained in the *m/z* range of 100–1500, and the MS1/MS2 resolution was set to 35 000/17 500.

### Targeted analysis using high‐performance liquid chromatography

The concentration of hydroxybenzoic acid, sinapic acid, ferulic acid, rutin, and kaempferol in *G. procumbens* leaves were determined using high‐performance liquid chromatography (HPLC) following a previously described method.^3^ For targeted metabolite analysis, 300 mg of lyophilized *G. procumbens* leaves were mixed with 5 mL of methanol containing 0.1% (*v/v*) phosphoric acid. The mixture was vortexed for 10 min, followed by sonication in an ice‐cooled ultrasonic bath for 60 min. After centrifugation at 12 000 × *g* for 10 min at 4 °C, the supernatant was filtered through a 0.45 μm membrane filter and injected into a Luna C18 column (250 mm × 4.6 mm, 5 μm; Phenomenex, Torrance, CA, USA) with an injection volume of 20 μL. The mobile phases consisted of 100% water (A) and 5% acetic acid in methanol (B), with a flow rate of 1 mL/min. Gradient elution was performed as follows: 0–10 min, 0–20% B; 10–20 min, 20–40% B; 20–30 min, 40–50% B; 30–40 min, 50–70% B; 40–50 min, 70–100% B; 50–75 min, 100–20% B. The eluted bioactive compounds were detected at 280 nm for phenolic acids and at 370 nm for flavonoids using an ultraviolet (UV)‐visible detector (UV‐2075 Plus; Jasco, Tokyo, Japan). For quercetin analysis, 0.1 g of the sample was dissolved in 40 mL of 60% ethanol and 5 mL of 6 N hydrochloric acid (HCl), followed by refluxing at 95 °C for 30 min. The solution was concentrated using a rotary vacuum evaporator, diluted to 50 mL with 60% ethanol, and filtered through a 0.45 μm membrane filter. The mobile phase consisted of water, 5% acetic acid, and acetonitrile (40:30:30, *v/v/v*) at a flow rate of 1 mL/min. The injection volume was 20 μL, and quercetin was detected at 370 nm using the same UV‐visible detector.

### Statistical analysis

Statistical analyses were performed using one‐way analysis of variance, followed by Tukey's *post hoc* test using SAS version 9.4 (SAS Institute Inc., Cary, NC, USA). MetaboAnalyst (https://www.metaboanalyst.ca; accessed on 1 March 2025) was used for principal component analysis (PCA).

## RESULTS AND DISCUSSION

### Effect of ultrasound treatment on antioxidant components and activities of *G. procumbens* leaves

**Figure 1:**
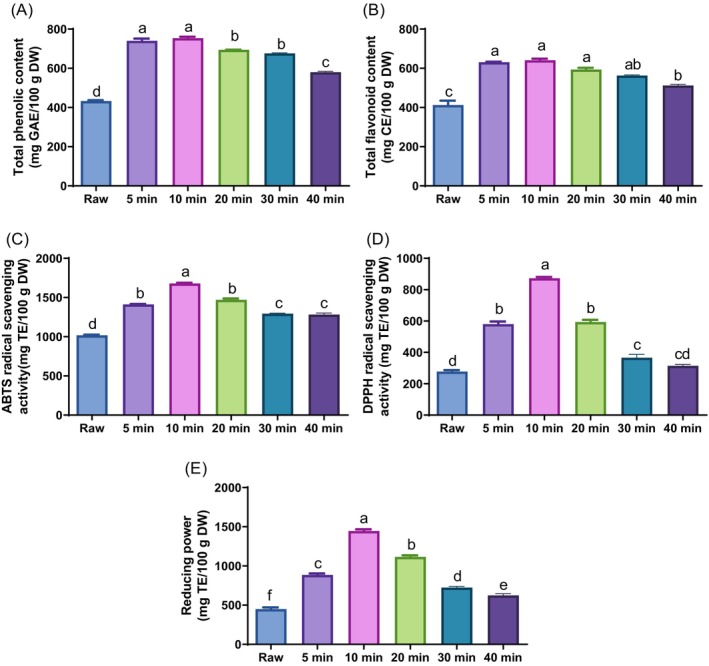
Effect of ultrasound‐treated *Gynura procumbens* (UTGP) on the total phenolic content (TPC), total flavonoid content (TFC), and antioxidant activities. TPC and TFC are expressed as milligrams of gallic acid equivalent per 100 g of dry weight (mg GAE/100 g DW) and milligrams of catechin equivalent per 100 g of dry weight (mg CE/100 g DW), respectively. The radical scavenging activity is expressed as milligrams of Trolox equivalent per 100 g of dry weight (mg TE/100 g DW). Different letters above the bars indicate significant differences at *P* < 0.05.

Several studies have shown that ultrasound treatment can trigger plant defense systems, leading to the enhancement of phytochemical levels.^3^, ^4^ To determine the optimal ultrasound treatment time, the leaves of *G. procumbens* were ultrasound treated for 5, 10, 20, 30, and 40 min, and the antioxidant components and activities were measured (Fig. 1). The TPC of the UTGP leaves was 433.3–753.4 mg gallic acid equivalent/100 g DW, while the TFC was 412.5–640.9 mg catechin equivalent/100 g DW. Compared to the other treatments, leaves treated to ultrasound for 10 min contained the highest antioxidant components. Moreover, the 10 min‐UTGP group significantly enhanced ABTS radical scavenging activity, DPPH radical scavenging activity, and reducing power by 1.7‐, 3.1‐, and 3.2‐fold, respectively, compared with the non‐treated leaves. Meanwhile, the antioxidant activities in the 20, 30, and 40 min‐treated leaves were significantly decreased compared with that in the 10 min‐treated leaves (ABTS, 1678.7 mg Trolox equivalent (TE)/100 g DW; DPPH, 832.9 mg TE/100 g DW; reducing power, 1446.0 mg TE/100 g DW). These decreases in antioxidant activity may be related to the reduction in flavonoid and phenolic contents. When plants are exposed to ultrasound conditions and produce high levels of secondary metabolites, they can trigger feedback mechanisms that increase enzyme levels to degrade these metabolites because plants have thresholds for the quantity of secondary metabolites that they can synthesize.^3^, ^21^ Therefore, 5, 10, and 20 min‐UTGP leaves were used in the subsequent experiments.

### Effect of ultrasound treatment on CAT, POD, TAL, and PAL activities in *G. procumbens* leaves

**Figure 2:**
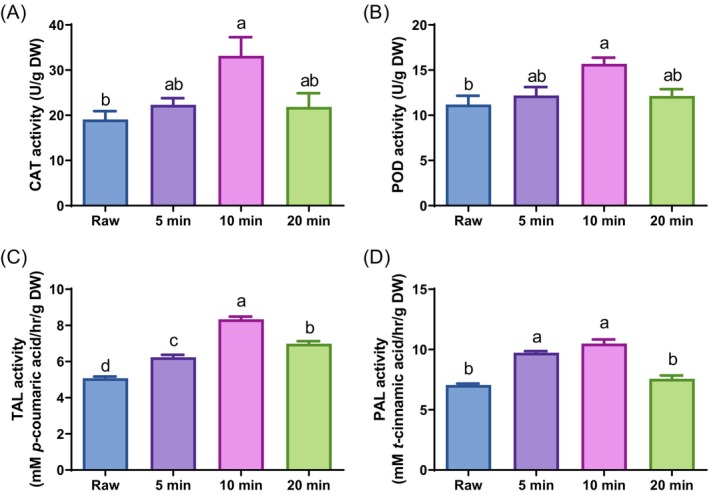
Effect of ultrasound‐treated *Gynura procumbens* (UTGP) on enzyme activities. (A) Catalase (CAT), (B) peroxidase (POD), (C) tyrosine ammonia‐lyase (TAL), and (D) phenylalanine ammonia‐lyase (PAL). Data are presented as the mean ± standard error (*n* = 3). Different letters above the bars indicate significant differences at *P* < 0.05.

Enzymes such as CAT, POD, TAL, and PAL play crucial roles in the biosynthesis and accumulation of phenolic acids and flavonoids in plants.^22^ PAL is a key enzyme in the phenylpropanoid pathway that catalyzes the conversion of phenylalanine to *trans*‐cinnamic acid – a precursor of various phenolic compounds.^23^ Elevated PAL activity is often associated with increased levels of phenolic acids and flavonoids, which enhance the antioxidant capacity of plants.^3^ Similarly, TAL contributes to the synthesis of *p*‐coumaric acid from tyrosine, which further promotes flavonoid production. CAT and POD are involved in the detoxification of ROS generated under stress conditions, indirectly influencing the accumulation of phenolic compounds.^24^ These enzymes support the accumulation of phenolic acids and flavonoids, which are vital for plant defense and contribute to the nutritional and medicinal properties of various plant species. To elucidate the underlying mechanisms responsible for the increased TPC and TFC in the 10 min‐UTGP leaves, various enzyme activities associated with the biosynthesis of phenolic acids and flavonoids in the plant were measured. The ultrasound treatment of *G. procumbens* leaves for 10 min significantly enhanced the CAT and POD activities compared to untreated *G. procumbens* leaves (Fig. 2(A),(B)). Moreover, the TAL and PAL activities of the 10 min‐UTGP leaves were markedly increased (Fig. 2(C),(D)).

Ultrasound is a type of physical energy source that produces a range of physical, chemical, and biological effects.^1^ Wu and Lin^25^ have reported that ultrasound treatment induces cross‐membrane ion fluxes, stimulates the production of ROS, and rapidly increases the PAL activity, followed by an increase in the production of polyphenols and phenolic compounds in the *Panax ginseng* cell suspension. Moreover, Nadar and Rathod^26^ have reported that ultrasound treatment is a promising technology for activating enzymes by altering their structural conformation. Seong *et al*.^3^ found that ultrasound treatment enhanced the activities of CAT and POD in *C. asiatica*, leading to an increased accumulation of various bioactive compounds, which is consistent with the current findings. These findings suggest that ultrasound treatment increases phenolic acid and flavonoid contents in *G. procumbens* leaves by enhancing the activities of CAT, POD, PAL, and TAL.

### Protective effect of UTGP leaves on H_2_O_2_ ‐induced muscle injury in C2C12 myoblasts and myotubes

**Figure 3:**
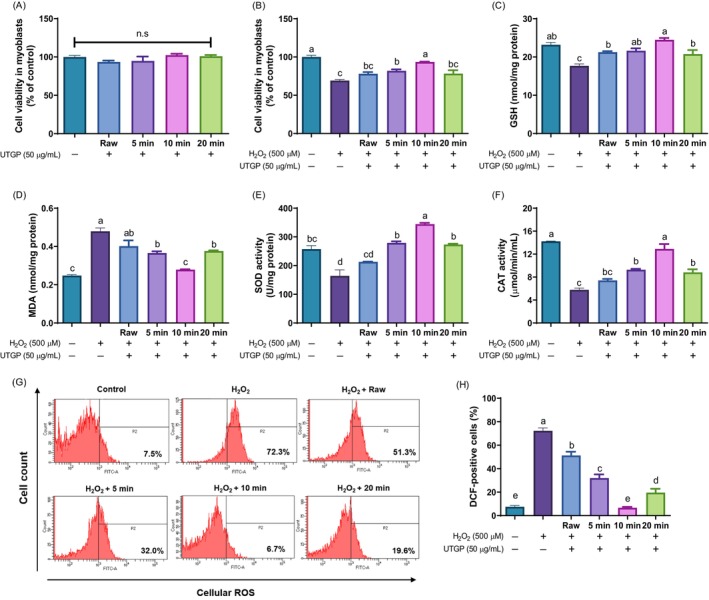
Effect of ultrasound‐treated *Gynura procumbens* (UTGP) on (A) cytotoxicity, (B) protective activity, (C) glutathione (GSH) level, (D) malondialdehyde (MDA) level, (E) superoxide dismutase (SOD) activity, (F) catalase (CAT) activity and (G, H) reactive oxygen species (ROS) production in hydrogen peroxide (H_2_O_2_)‐induced C2C12 myoblasts. Data are presented as the mean ± standard error (*n* = 3). Different letters above the bars indicate significant differences at *P* < 0.05.

In this study, the protective effects of UTGP leaves against oxidative stress induced by H_2_O_2_ in C2C12 myoblasts and myotubes were evaluated. Prior to the protective effect assay, the cytotoxicity of UTGP leaves (25, 50, and 100 μg/mL) and H_2_O_2_ (400–700 μmol/L) was examined in C2C12 myoblasts (Supporting Information, Fig. S1). The results demonstrated that treatment with UTGP did not significantly affect the viability of the myoblasts under normal conditions, indicating its safety at the tested concentrations, whereas cell viability decreased to approximately 70% at H_2_O_2_ concentrations of 500 μmol/L and higher (Figs S1 and 3(A)). Therefore, 50 μg/mL of UTGP, representing the middle concentration within the non‐toxic range, and 500 μmol/L of H_2_O_2_, the optimal concentration to induce oxidative stress, were selected for subsequent experiments. As shown in Fig. 3(B), treatment with UTGP extracts enhanced cell viability by 12.8% (untreated), 21.2% (5 min‐treated), 35.3% (10 min‐treated), and 7.0% (20 min‐treated), compared to H_2_O_2_‐treated cells. Various oxidative stress markers were assessed in addition to cell viability. The levels of GSH were significantly elevated in UTGP‐treated myoblasts, particularly in the 10 min‐UTGP, compared with the untreated and other treatment durations (Fig. 3(C)). This increase in the levels of GSH indicates an enhanced antioxidant defense mechanism that is critical in combating oxidative stress. Conversely, the levels of MDA – which is a marker of lipid peroxidation – were significantly lower in myoblasts treated with 10 min‐UTGP (Fig. 3(D)). Moreover, SOD and CAT activities were significantly higher in myoblasts treated with UTGP, particularly after 10 min of ultrasound treatment (Fig. 3(E),(F)). The flow cytometry data shown in Fig. 3(G),(H) illustrate the relative levels of ROS production under various experimental conditions. Treatment with H_2_O_2_ significantly increased ROS production, with 72.3% of the cell population displaying elevated ROS levels. In contrast, UTGP treatment reduced ROS levels, compared to the H_2_O_2_ treatment. Notably, the 10 min‐UTGP treatment resulted in ROS levels similar to those of the control group.

**Figure 4:**
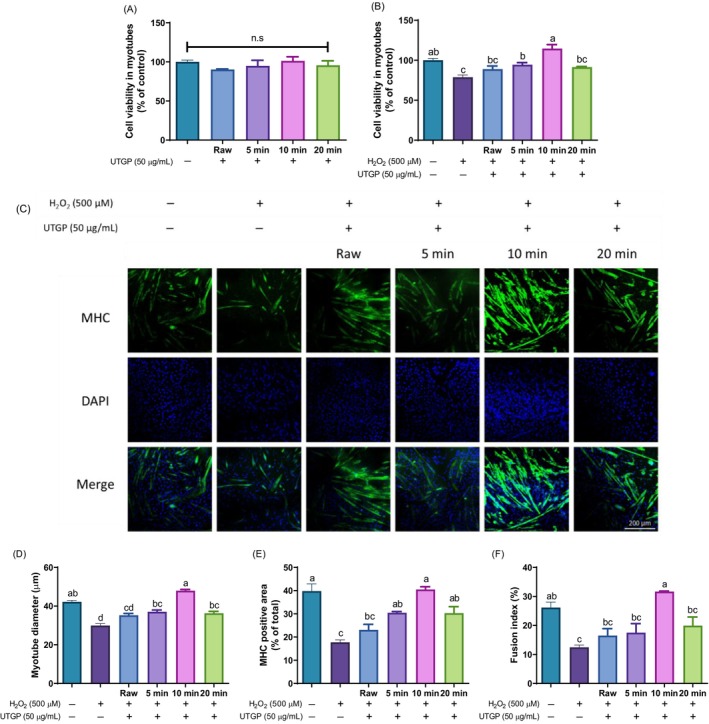
Effect of ultrasound‐treated *Gynura procumbens* (UTGP) on (A) cytotoxicity, (B) protective activity, (C) myosin heavy chain (MHC) immunofluorescence staining images (MHC: green and DAPI: blue), (D) myotube diameter, (E) MHC‐positive area, and (F) fusion index in hydrogen peroxide (H_2_O_2_)‐induced C2C12 myotubes. Data are presented as the mean ± standard error (*n* = 3). Different letters above the bars indicate significant differences at *P* < 0.05.

The effects of UTGP on skeletal muscle differentiation were further investigated; no cytotoxicity was observed in C2C12 myotubes (Fig. 4(A)). The results showed that, compared to H_2_O_2_‐treated cells, the cell viability was enhanced for all ultrasound treatments, with 10 min‐UTGP exhibiting the most pronounced protective effect (Fig. 4(B)). Immunofluorescence staining revealed that the myotube diameter and MHC positive area were considerably increased in 10 min‐UTGP, indicating enhanced muscle differentiation (Fig. 4(C)–(E)). The fusion index was also significantly elevated in this group, suggesting that it promotes myoblast fusion (Fig. 4(F)).

To the best of our knowledge, this is the first study to demonstrate that *G. procumbens* leaves can alleviate muscle injury and enhance muscle differentiation. Moreover, ultrasound treatment altered the effects of *G. procumbens* leaves on muscle protection and differentiation of C2C12 myoblasts and myotubes. This may be due to changes in the content of the functional compounds in the UTGP leaves. *Gynura procumbens* leaves contain several potential antioxidant components,^12^ which are well known for their ability to prevent muscle atrophy.^27^ Flavonoids and phenolic acids have been reported to exert beneficial effects on muscle health.^28^, ^29^ Chen *et al*.^30^ demonstrated that quercetin can reduce dexamethasone‐induced mitochondrial malfunction in skeletal muscle cells. Hour *et al*.^31^ showed that quercetin induces differentiation and improves muscle regeneration. These findings highlight that ultrasound treatment not only enhances the biological activity of *G. procumbens* leaves by elevating the levels of flavonoids and phenolic acids but also increases their therapeutic potential in improving muscle‐related diseases.

### Profiling of metabolite changes induced by ultrasound treatment through non‐targeted and targeted metabolomics

**Figure 5:**
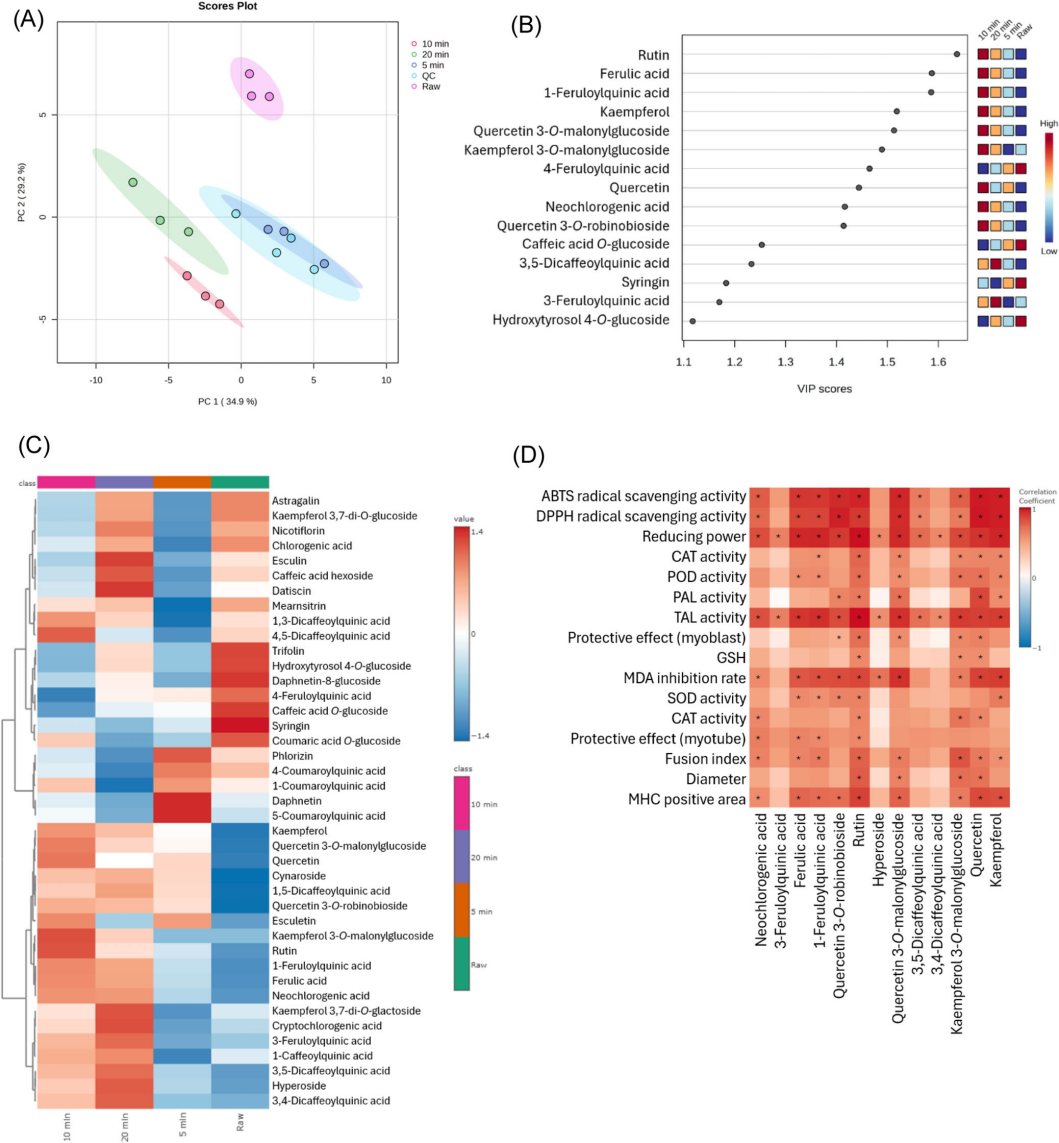
Principal component analysis (PCA) scores plot (A), variable importance in projection (VIP) scores (B), heatmap (C), and correlation between metabolites and biological activities (D). *P < 0.05.

Non‐targeted metabolomic analyses have been employed in numerous studies to assess the changes in metabolites, including those focused on phytochemical profiling.^32^ We conducted a non‐targeted metabolomic analysis to identify the specific components responsible for the protective effects in myoblasts and myotubes. In this study, 41 metabolites were identified by non‐targeted analysis. PCA demonstrated that the UTGP samples within each group exhibited tight clustering, and the four groups were distinctly separated, indicating significant metabolic differences (Fig. 5(A)). In PCA, principal component one (PC1) and principal component two (PC2) accounted for 34.9% and 29.2% of the variance, respectively, confirming the separation among the ultrasound treatment times. The quality controls were densely clustered around (0,0), indicating consistent and reliable experimental performance. Rutin, ferulic acid, 1‐feruloylquinic acid, and kaempferol showed high variable importance in projection (VIP) scores of 1.64, 1.59, 1.59, and 1.52, respectively. The 10 min‐UTGP sample showed the highest levels of these metabolites, followed by the 20 min‐UTGP sample (Fig. 5(B)). Figure 5(C) shows a clear visualization of the relative abundance of metabolites in UTGP across different ultrasound treatment times. After 10 min of ultrasound treatment, the content of kaempferol 3‐*O*‐malonylglucoside increased significantly compared to untreated leaves, whereas the level of astragalin showed a sharp decrease. These results suggest that ultrasonic treatment may influence the metabolic pathway of kaempferol derivatives. Kaempferol is converted to astragalin by glucosyltransferase and subsequently transformed into kaempferol 3‐*O*‐malonylglucoside by malonyltransferase. The marked increase in kaempferol 3‐*O*‐malonylglucoside and the rapid decrease in astragalin after 10 min of ultrasound treatment suggest that ultrasonic treatment may regulate the activity of glucosyltransferase and malonyltransferase, facilitating the rapid malonylation of astragalin. Similarly, quercetin is converted to isoquercitrin by glucosyl transferase, followed by conversion to quercetin 3‐*O*‐malonylglucoside by malonyl transferase.^33^ Thus, ultrasound treatment possibly activates both glucosyltransferase and malonyltransferase, thereby increasing the accumulation of quercetin 3‐*O*‐malonylglucoside and kaempferol 3‐*O*‐malonylglucoside.

To predict the bioactive compounds responsible for antioxidant and muscle protective effects, a correlation analysis was conducted between the metabolites of UTGP leaves and their biological activities. Correlation analysis revealed that among the metabolites of UTGP with a VIP score > 1.0, 13 compounds exhibited significant positive correlations (*P* < 0.05) with antioxidant activities, muscle protection, and differentiation markers. Notably, rutin, quercetin, kaempferol 3‐*O*‐malonylglucoside, quercetin 3‐*O*‐malonylglucoside, kaempferol, 1‐feruloylquinic acid, and ferulic acid were identified as the key contributors to the bioactive potential of the 10 min‐UTGP leaves (Fig. 5(D)).

To confirm the concentrations of these metabolites, we performed targeted analysis in UTGP leaves, excluding standards that were not commercially available (Table 1). The results showed that 10 min‐UTGP leaves had the highest concentrations of quercetin (13.52 ± 1.06 mg/100 g DW), rutin (46.58 ± 0.49 mg/100 g DW), and kaempferol (4.10 ± 0.39 mg/100 g DW). Unlike the untargeted analysis, ferulic acid was not detected in the targeted analysis, while only sinapic acid and *p*‐hydroxybenzoic acid were identified, emphasizing the importance of targeted approaches for accurate compound identification. Sinapic acid levels remained consistent across different ultrasound treatment times, whereas hydroxybenzoic acid content increased from 375.15 ± 10.04 mg/100 g DW (0 min) to 443.07 ± 12.86 mg/100 g DW (5 min), peaked at 745.01 ± 8.77 mg/100 g DW (10 min), and slightly decreased to 719.00 ± 18.49 mg/100 g DW at 20 min. Among these, quercetin is particularly noteworthy, as it has been reported to suppress the expression of specific atrophic factors, such as Atrogin‐1 and MuRF1, in the skeletal muscle of high‐fat diet (HFD)‐fed obese mice, thereby protecting against muscle mass loss and reduction in muscle fiber size.^34^ Another study has shown that rutin alleviates dexamethasone‐induced muscle loss in C2C12 myotube and C57BL/6 mice through regulating FOXO3 signaling pathway.^35^ Moreover, kaempferol has beneficial effects on improvement of muscle atrophy via enhancing Nrf2 signaling pathway in mice.^36^ In contrast, the effect of hydroxybenzoic acid on muscle atrophy has not yet been elucidated. Taken together, these findings suggest that ultrasonic treatment enhances the accumulation of key bioactive compounds, such as quercetin, rutin, and kaempferol, which may contribute to the antioxidant and muscle‐protective effects of UTGP through modulation of muscle atrophy‐related signaling pathways.

**Table 1:** Determination of bioactive compounds in ultrasound‐treated *Gynura procumbens* (UTGP) leaves

	Ultrasound treatment time			
	0 min (Raw)	5 min	10 min	20 min
*Flavonoids (mg/100 g DW)*				
Quercetin	6.46 ± 0.41^c^	9.64 ± 0.33^b^	13.52 ± 1.06^a^	12.53 ± 0.76^a^
Rutin	21.93 ± 0.67^d^	36.01 ± 0.10^c^	46.58 ± 0.49^a^	42.85 ± 0.75^b^
Kaempferol	1.38 ± 0.19^c^	2.68 ± 0.13^b^	4.10 ± 0.39^a^	2.56 ± 0.09^b^
*Phenolic acids (mg/100 g DW)*				
Hydroxybenzoic acid	375.15 ± 10.04^c^	443.07 ± 12.86^b^	745.01 ± 8.77^a^	719.00 ± 18.49^a^
Sinapic acid	5.10 ± 0.38^a^	5.80 ± 0.37^a^	6.25 ± 0.55^a^	5.35 ± 0.29^a^
Ferulic acid	n.d.	n.d.	n.d.	n.d.

*Note*: Each value is expressed as the mean ± standard deviation (*n* = 3). Values within a row followed by different superscript letters are significantly different according to Tukey's test (*P* < 0.05). DW, dry weight; n.d., not detected.

## CONCLUSION

The 10 min‐ultrasound treatment significantly increased the concentration of bioactive compounds, including quercetin, rutin, and kaempferol, resulting in enhanced antioxidant capacity and biological activity of *G. procumbens* leaves. Therefore, postharvest ultrasound treatment represents a promising approach for enhancing the bioactive properties of medicinal plants, with potential applications in muscle health management and oxidative stress‐related conditions. *Gynura procumbens* can be processed into functional foods or nutraceuticals in the form of extracts, powders, or capsules. Postharvest ultrasound treatment offers a dual benefit by improving the removal of surface contaminants through washing and simultaneously enhancing the accumulation of functional phytochemicals. This approach highlights the practical potential of UTGP for development as a value‐added functional or medicinal product with commercial and therapeutic applications. However, to better understand the underlying molecular mechanisms, a comprehensive transcriptomic analysis is required to systematically assess the expression of all enzymes involved in the biosynthetic pathways of phenolic acids and flavonoids. This approach will provide deeper insight into how ultrasound treatment modulates secondary metabolite biosynthesis at the transcriptional level. In addition, future research should include *in vivo* studies on muscle atrophy models to clarify the mechanism of action.

## FUNDING INFORMATION

This research was supported by Basic Science Research Program through the National Research Foundation of Korea (NRF) funded by the Ministry of Education (RS‐2024‐00450823).

## CONFLICT OF INTEREST

The authors declare no competing interests.

## Supporting information


**Figure S1.** Cytotoxicity of ultrasound‐treated *Gynura procumbens* (UTGP) leaf extracts and optimization of H_2_O_2_ concentration in C2C12 myoblasts. Cell viability was measured after treatment with UTGP leaf extracts at concentrations of (A) 25 μg/mL, (B) 50 μg/mL, and (C) 100 μg/mL. (D) C2C12 cells were exposed to different concentrations of H_2_O_2_ (400–700 μmol/L) for 24 h to determine an appropriate oxidative stress condition. Data are presented as mean ± SD (*n* = 3). ^##^ *P* < 0.01, ^####^ *P* < 0.0001 *versus* untreated control; n.s., not significant.

## Data Availability

The data that support the findings of this study are available from the corresponding author upon reasonable request.
