# Supplementary material for: Effect of post‐harvest ultrasound treatment on phytochemical enhancement in Gynura procumbens leaves and their protection against oxidative stress‐induced muscle atrophy
Source: J Sci Food Agric. 2025 Nov 20;106(4):2270–9. doi: 10.1002/jsfa.70335 (PMC12872237; doi:10.1002/jsfa.70335)
Supplement: Supplementary file 1 — Figure S1. Cytotoxicity of ultrasound‐treated Gynura procumbens (UTGP) leaf extracts and optimization of H2O2 concentration in C2C12 myoblasts. Cell viability was measured after treatment with UTGP leaf extracts at concentrations of (A) 25 μg/mL, (B) 50 μg/mL, and (C) 100 μg/mL. (D) C2C12 cells were exposed to different concentrations of H2O2 (400–700 μmol/L) for 24 h to determine an appropriate oxidative stress condition. Data are presented as mean ± SD (n = 3). ## P < 0.01, #### P < 0.0001 versus untreated control; n.s., not significant. [file JSFA-106-2270-s001.docx]

**Supporting data**

**Figure S1. Cytotoxicity of ultrasound-treated *Gynura procumbens* (UTGP) leaf extracts and optimization of H₂O₂ concentration in C2C12 myoblasts.** Cell viability was measured after treatment with UTGP leaf extracts at concentrations of (A) 25 μg/mL, (B) 50 μg/mL, and (C) 100 μg/mL. (D) C2C12 cells were exposed to different concentrations of H₂O₂ (400–700 μM) for 24 h to determine an appropriate oxidative stress condition. Data are presented as mean ± SD (n = 3). ^##^p < 0.01, ^####^p < 0.0001 vs. untreated control; n.s., not significant.
